# Evaluating Slow Pyrolysis of *Parthenium hysterophorus* Biochar: Perspectives to Acidic Soil Amelioration and Growth of Selected Wheat (*Triticum aestivum*) Varieties

**DOI:** 10.1155/2022/8181742

**Published:** 2022-01-04

**Authors:** Meseret Muche, Eyayu Molla, Sultan Mohammed, Esubalew Sintie, Ahmed Hassen

**Affiliations:** ^1^Department of Biology, Woldia University, P.O. Box 400, Woldia, Ethiopia; ^2^Department of Natural Resource Management, College of Agriculture and Environmental Sciences, Bahir Dar University, Bahir Dar, Ethiopia

## Abstract

Application of biochar on acidic soils may improve soil fertility and crop productivity. This study aimed to explore the relevance of parthenium biochar-induced changes in the physicochemical properties and agronomic performance of the selected wheat varieties in acidic soils. A pot trial was used in determining the effect of slow pyrolysis parthenium biochar on acidic soils and the agronomic performance of wheat varieties. A general linear model (GLM) of multivariate analysis and principal component analysis (PCA) was used to compare functional variation among soil assayed parameters with biochar dosages and years. Biochar-treated acidic soils did not show significant differences in their physical properties. However, a significant incremental trend was observed in the soil moisture content. The biochar-amended acidic soils showed noticeable differences in the soil pH, available phosphorous, and exchangeable bases (Ca, K, and Na) compared to the control. In all soil samples, a decreasing trend in the soil micronutrients was observed with an increase in the biochar amounts. The analysis also unveiled significant changes in root length, root and shoot dry biomass, and plant height of wheat varieties in response to the biochar amendments. The application of 19.5 t/ha and 23 t/ha dosages of biochar gave the maximum changes in the agronomic performance of *Kekeba* and *Ogolcha* varieties, while the minimum was obtained in the 26.5 t/ha and the control. Furthermore, PCA axis 1 accounted for 74.34% of the total variance within a higher eigenvector value (10.4076), and most of the soil parameters were positively correlated with CEC (0.29), available phosphorous (0.29), and soil pH (0.28); however, the micronutrients were negatively correlated. In conclusion, *Parthenium hysterophorus* biochar has the potential to amend acidic soils, and thus, the application of 16.0, 19.5, and 23 t·ha^−1^ biochar dosages are considered suitable to reduce the soil acidity level and improve the agronomic performance of wheat varieties. However, extensive research will be needed to determine the effects of biochar on soil properties and crop production in field conditions.

## 1. Introduction

In Ethiopia, low soil fertility is one of the factors limiting the yield of many crops. This is caused by the removal of surface soil by erosion, crop removal of nutrients from the soil, total removal of plant residue from farmland, and lack of proper crop rotation practices [1, 2]. On the other hand, invasive herbaceous weedy species such as *Parthenium hysterophorus* are increasing in different regions of Ethiopia [3]. *P. hysterophorus* (Asteraceae) is an aggressive alien weed species native to the Americas, and at present, it is extensively spread in Asia, Australia, and Africa [4] and Ethiopia [3]. It grows along roadsides and in fallow and cultivated lands, riverbanks, disturbed areas, and floodplains. It competes with and replaces native species and is also a significant crop weed [3–5]. It is the most noxious weed in the agricultural system due to prolific seed production, allelopathic effect, and competitiveness [5]. Various studies have been conducted on the toxic effect of the *P. hysterophorus* on a wide range of crops. For instance, studies showed that extracts of the weed at lower concentrations (2.5–4%) significantly diminished seed germination, seedling biomass, and chlorophyll content of wheat by 60–75% [6, 7] and grain yields of sorghum and maize by 40–97% and 30–60%, respectively [5]. However, different studies proposed that parthenium biochar can be used as green manure, compost, biocontrol, and soil amelioration that improve the soil physical, chemical, and biological properties and is a source of readily available plant micro- and macronutrients [8–10]. Hence, amendment of soil acidity through pyrolysis carbon extracted biochar from this aggressive weed (*P. hysterophorus*) is momentous. Biochar (BC) is a fine-grained carbon-rich product obtained when the biomass is heating in an oxygen-depleted atmosphere [11, 12]. It contains porous carbonaceous and an array of functional groups [13]. Recently, the potential of biochar use to recapture excess soil nutrients, crops, and remove contaminants has received growing attention [14–17]. The feedstock sources and pyrolytic temperatures are the principal factors for the nutrient provisions. And thence, the herbaceous feedstock may pyrolyze above 400°C and woody raw materials even above 800°C [18]. The addition of biochar to acidic soils changes soil pH from 3.9 to 5.1 [19], boost electrical conductivity [20], boost cation exchange capacity from 7.41 to 10.8 cmol^+^/kg [21], and increase the percent base saturation from 6.4 to 26% and modifies soil acidity [22]. Therefore, the present study was initiated to assess the potential effect of *P. hysterophorus* biochar on the amelioration of acidic soils and the agronomic performance of selected wheat (*Triticum aestivum*) crop varieties.

## 2. Materials and Methods

### 2.1. Description of the Study Area

The study was conducted at the research site of Woldia University, Northeastern Ethiopia, from June 2018 to November 2020. Geographically, the site lies between 11°35′ and 12°00′ N latitude and 39°14′ and 39°48′ E longitude and 2,740 meters above sea level (masl). The mean annual rainfall recorded during the study period was 1,050 mm, and the average annual minimum and maximum temperatures were 18 and 28.7°C, respectively [23]. The district was a representative of wet highland and characterized by erosion-prone, susceptible to acidity, low potential, and oxen plow cereal belt area. The main crop types grown in the district are oats (*Avena sativa* L.), line seed (*Linum usitatissimum* L.), barley (*Hordeum vulgare* L.), and wheat (*T. aestivum*). The area was selected for the study because soil acidity and invasion of parthenium weed in the area are important issues that require urgent attention.

### 2.2. Preparation of the Biochar Component

Fresh *P. hysterophorus* weed was collected in the vicinity of the study site before flowering to prevent seed dispersal. The entire plant material (biomass) was washed with distilled water to remove impurities and allowed to air-dry. The dried weed was cut down into smaller pieces (10–15 cm) and treated with an active chemical (7% H_2_SO_4_/1 kg) to lower the temperature of carbonization [24]. A kilogram of chemically treated dried sample was tightly placed in a closed perforated austenitic stainless steel covered with a fitting lid and inserted into the muffle furnace and then charred at 350°C (slow pyrolysis temperature) for 30 minutes in an oxygen-free medium [25]. The biochar produced was transferred from the muffle furnace into the pot (height of 40.0 cm and 27.0 cm width) and washed thoroughly with distilled water to remove the component of chemically active acid. At the end, the recommended dosages of fine-grained biochar was characterized (Table 1; Supplementary 1), measured [26], and denoted based on the dose of parthenium biochar (PB) as PB0% (0 t/ha), PB5.33% (12.5 t/ha), PB8.0% (16.0 t/ha), PB10.67% (19.5 t/ha), PB13.3% (23 t/ha), and PB16% (26.5 t/ha). The pyrolyzed biochar was later grounded and sieved with a 0.05 mm sieve and made ready for application.

### 2.3. Research Design, Soil Analysis, and Crop Data Collection

#### 2.3.1. Treatments and Experimental Design

In this study, a pot experimental design that involves a complete randomized design (CRD) with three replications of three (wheat varieties) × six (biochar rates) factorial combinations was used. Before the experiment, the pH of the soil samples (ranging from 5.2 to 5.6) was randomly collected at 0–25 cm depth from 15 pits of acidic farmlands. The soil samples were bulked together to serve as composite soil samples. Then a 12 kg of dry acidic soil was mixed with different dosages of biochar filled in each 40 cm height and 27.0 widths experimental pot. The *P. hysterophorus* biochar dosages were 0, 12.5, 16.0, 19.5, 23, and 26.5 t/ha, which accounted for the dry weights of the potting soil (Supplementary 2). The pots were left for two months with three days interval of 2000 mL watering for decomposition and merely mixed the biochar into the acidic soil [26, 27]. After preparing the pots filled with equal amounts of soil, different amount of biochar was added as per treatments in a complete randomized design with three replications. Three wheat varieties (*Kekeba*, *Ogolcha*, and *Kingbird*) obtained from Ethiopia Agricultural Research Institute were used as test crops. Before sowing, the seeds were first washed with distilled water and sterilized with 2% sodium hypochlorite for 2 minutes. Accordingly, pots were filled with equal amounts of soil and different dosages of biochar and arranged into blocks, and one of the treatments was used as a control (Supplementary 2). Afterward, 15 viable seeds were selected and evenly sowed into each pot, and each germinating pot was regularly supplied with 2,000 mL of water once a day in the morning. Cultural practices such as weeding, hoeing, disease, and pest control were applied uniformly for all treatments to produce healthy and pure seedlings. Weeds were managed by hand weeding after weed emergence. Finally, the wheat varieties were separately harvested.

#### 2.3.2. Soil Analysis

Soil samples were collected before and after treatments (Table 1) in the farmlands and analyzed following the standard laboratory protocols. Soil particle size was analyzed following the hydrometer or Bouyoucos method [28]. Soil moisture content was determined by the percentage weight loss of the soil sample after being dried at 105°C divided by the dry soil weight [29]. The soil pH was measured in water (pH (H_2_O)) and potassium chloride (1 M KCl) [28]. Soil organic carbon content was analyzed by wet combustion or dichromate oxidation methods [30]. The soil available phosphorous content was determined by the 0.5 M sodium bicarbonate extraction solution/pH 8.5/method of Olsen as described by [20]. Exchangeable basic cations (Ca^2+^, Mg^2+^, K^+^, and Na^+^) were analyzed by saturating the soil samples with 1 N NH_4_OAc solution at pH 7. Then Ca^2+^ and Mg^2+^ were determined from the extract using atomic absorption spectrometry (AAS), while exchangeable K^+^ and Na^+^ were determined using a flame photometer from the same extracted [31]. Cation exchange capacity (CEC) was estimated titrimetrically by distillation of ammonium displaced by sodium from NaCl solution [32]. Furthermore, the available micronutrients (Cu, Mn, Zn, and Fe) were measured after extraction with 1 M NH_4_OAc as described by [31].

#### 2.3.3. Agronomic Data Collection

The following phonological crop data were collected in each wheat variety as recommended by [33]. The assay parameters were plant height (PH), head length (HL), spike number (SN), seed number per plant (SNPP), root length (RL), shoot dry biomass (SDB), and root dry biomass (RDB). Plant height (PH; cm) was recorded from randomly selected ten wheat plants and measured from the soil surface to the top-most growth point of plants at the time of physiological maturity, and the mean value was used for analysis. Head length (HL; cm) was recorded from randomly selected ten wheat plants from the uppermost part of the peduncle to the tip of grain-bearing parts at maturity, and the mean value was taken for analysis. Spikes number (SN) was counted from ten randomly taken wheat plants at physiological maturity in length, and then average value was recorded. Seed number per plant (SNPP) was measured by counting the number of grains per spike in each experimental pot at harvesting time. Root length (RL; cm) was measured lengthwise from the crown (underneath the ground where the secondary roots emerge) to the tip of the primary root at harvesting time after properly uprooted from the experimental pot. Shoot dry biomass (SDB; gram) was measured from ten randomly selected wheat plants from the net pot area at the time of harvesting, and then the samples were air-dried for 72 hours, after which weight was taken. Root dry biomass (RDB; gram) was also recorded by taking the average below-ground biomass of ten randomly selected wheat plants after the samples were air drying out for 72 hours.

### 2.4. Data Analysis

The data were subjected to multivariate analysis (two- and three-way ANOVA) using the general linear model (GLM) procedures of SAS v.9.1.3 to compare the soil physicochemical properties and growth of the varieties influenced by parthenium weed biochar across the soil sample years. Mean comparisons were employed using least significant difference (LSD) at 5% levels. The principal component analysis (PCA) was used for determining the functional variation of the soil assayed parameters after the data log-transformed using PAST version 3.0 statistical analysis software.

## 3. Results

The biochar made from the *P. hysterophorus* weed improves the soil quality and increases the growth of the selected wheat varieties. Thus, parthenium biochar is characterized by higher pH, C content, exchangeable bases, and available phosphorus content (Table 1), which amends acidity in the soil system.

### 3.1. Influence of *P. hysterophorus* Biochar on the Physical Properties of Acidic Soil

Soil particle distribution did not show a significant (*p* > 0.05) difference between the soils sampled in different years (Table 2). Similarly, there was no significant difference between the interaction effect of sand (*F* (5, 35) = 2.3; *p* > 0.05; *R*^2^ = 0.84), clay (*F* (5, 35) = 1.7; *p* > 0.05; *R*^2^ = 0.72), and silt particles contents (*F* (5, 35) = 2.1; *p* > 0.05; *R*^2^ = 0.52; Table 2). Conversely, there was an increase in the clay (*p* ≤ 0.001) and silt (*p* ≤ 0.05) contents. However, a significant reduction in the sand fraction (*p* ≤ 0.001) was observed with an increase in the dose of mixed biochar (Tables 2 and 3). The post hoc test in Table 3 revealed a higher overall mean of the sand fraction (61.3 ± 1.0) was recorded in the control (0 t/ha), and however, the clay (23.0 ± 0.7) and silt (20.5 ± 0.00) contents were observed in 16.0 t/ha and 26.5 t/ha biochar rates, respectively. The soil moisture content (SMC) showed a consistent and significant (*p* ≤ 0.01) change among the soils treated with different dosages of *P. hysterophorus* biochar and soil sample years (*p* ≤ 0.05). However, the interaction effect (*F* (5, 35) = 1.98; *p* > 0.05; *R*^2^ = 0.95) between the soil sample years and biochar application was insignificant (Table 2). Irrespective of the interaction effect, the soil water content was increasingly higher as biochar amounts increased to result in a slight increase in the overall mean soil moisture content (21.6 ± 0.5) in the soil with a 26.5 t/ha (16%) *P. hysterophorus* biochar dose compared to the control (15.5 ± 0.5; Table 3).

### 3.2. Chemical Properties of Acidic Soil Treated by *P. hysterophorus* Biochar

Soil pH showed a statistically significant difference (*p* ≤ 0.01) between the biochar dosages across the soil sample years (Table 4). Regardless of the interaction effects, there was an increasing trend in the soil pH values with the pyrolysis weed (Table 5). The highest soil pH (H_2_O: 7.8 ± 0.5 and KCl: 7.1 ± 0.5) was recorded in the soil treated with 26.5 t/ha biochar, while the lowest was observed in the control (Table 5). In addition, a significant (*p* ≤ 0.001) increase in EC content occurred as biochar concentration increased within the soil sample years (*F* (5, 35) = 10.07; *p* ≤ 0.001; *R*^2^ = 0.97; Table 5). Application of a 26.5 t/ha of biochar increased the soil EC by 14.88 and 12.33% as compared to the control in the first and second soil sample years, respectively. Similarly, biochar-treated acidic soils across the soil sample years showed significant (*p* ≤ 0.001) changes in exchangeable bases (Ca, K, and Na) and CEC (Table 4). In addition, there was a significant interaction effect between exchangeable Ca^++^ (*F* (5, 35) = 5.8; *p* ≤ 0.005; *R*^2^ = 0.90), Na^+^ (*F* (5, 35) = 5.4; *p* ≤ 0.005; *R*^2^ = 0.81), and CEC (*F* (5, 35) = 31.4; *p* ≤ 0.005; *R*^2^ = 0.92; Table 4). The higher overall average values of the exchangeable bases and CEC found in the *P*. *hysterophorus* biochar ranged between 23 and 26.5 t/ha (Table 5). On the contrary, the exchangeable Mg^2+^ content was not influenced by the biochar dosages (*p* > 0.05) and with the interaction effect (Table 4). Despite the interaction effect, a maximum (7.3 ± 2.0 cmol^+^/kg) value was recorded in the soil treated with 16 t/ha of biochar and the minimum in control (Table 5). The contents of available phosphorous and soil organic carbon also showed a considerable increase with the increase in biochar amounts and soil sample years (Table 4). As a result, the highest Av. P (73.2 ± 9.2 ppm) and SOC (2.2 ± 0.5%) contents found in the soils treated with 26.5 t/ha biochar that raised the Av. P content up to 5.75% and SOC by 3.6% compared to the control (Table 5).

**Figure 1:**
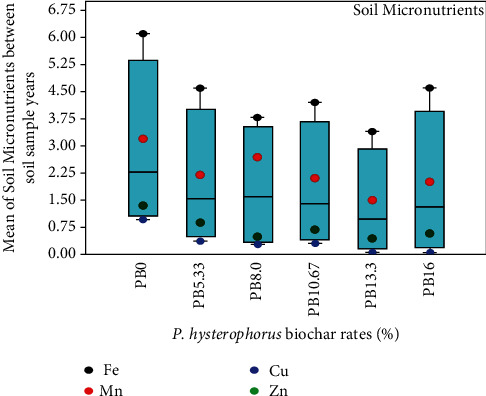
The effect of *P. hysterophorus* biochar on soil micronutrients of acidic soil between two soil sampling years.

The multivariate analysis also revealed a significant difference (*p* ≤ 0.001) in the contents of soil micronutrients between biochar-treated soils, but not in the interaction effect of the soil sample years and the amounts (Table 6). Irrespective of the significant difference in the interaction effect, there was a consistent decrease in the amounts of micronutrients with increasing parthenium biochar dosages from 12.5 t/ha to 23 t/ha (Figure 1). However, the highest average values of Fe (6.0 ± 0.6), Cu (0.96 ± 0.5), Mn (3.2 ± 0.3), and Zn (1.3 ± 0.2) registered in soils amended with no biochar (Figure 1).

### 3.3. Variation of the Soil Chemical Properties with Integrated Treatments

**Figure 2:**
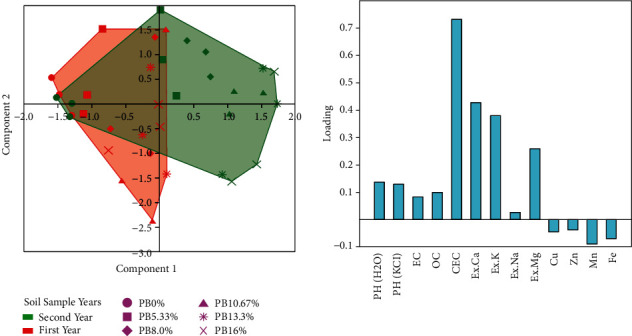
Biplot PCA chart indicating soil chemical properties of acidic soil as influenced by the parthenium biochar across years. The soil characteristics used were pH, EC, % OC, CEC, Ex.Ca, Ex.K, Ex.Mg, Ex.Mg, Cu, Zn, Mn, and Fe.

The amendment potential of different dosages of biochar on the chemical properties of acidic soils in the two years' time was evaluated using multivariate analysis. PCA axis 1 accounted for 74.34% variance and total eigenvalue equivalence to 10.407 and positively correlated with the soil EC (0.294), CEC (0.291), and available phosphorus (0.289) and negatively loading with the micronutrients including Mn (−0.27) and Zn (−0.25) (Table 7; Figure 2). The PCA biplot revealed the soil sampling years (first and second) and biochar dosages. Thus soil sample year two has a high degree of association with the biochar dosages, apart from the control treatments that had no significant changes across soil sampling years owning to no parthenium biochar (Figure 2). In contrast, the soil sample year one and biochar dosages did not show a higher association. However, the availability of those parthenium biochar-induced nutrients enhanced along with years within decreasing trends of the micronutrients.

### 3.4. Effect of Biochar on the Agronomic Performance of Wheat Varieties

**Figure 3:**
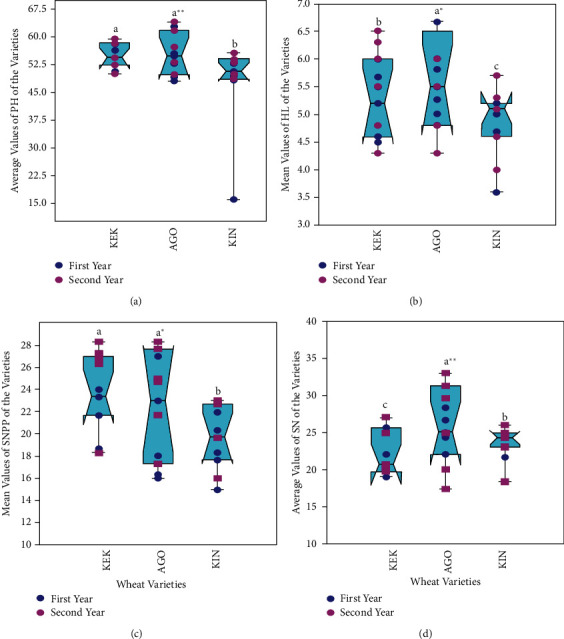
The agronomic performance of wheat varieties (KEK, AGO, and KIN) as influenced by different parthenium biochar and wheat growing years.

The multivariate analysis revealed that plant height (PH) and head length (HL) of wheat varieties were significantly (*p* ≤ 0.01; *p* ≤ 0.05) affected by the application of different biochar during the first and second years growth period (Table 8). There were significant differences (*p* ≤ 0.001) in seed number per plant (SNPP) and spikelet numbers (SN) among the wheat varieties between biochar amendments across both years (Table 8). Despite the influences in the biochar rates, there was no interaction effect among the treatment factors. The analysis further indicated that *Ogolcha* (AGO) wheat variety showed the highest performance in PH (62.67 ± 4.6 cm), HL (6.5 ± 0.4 cm), SNPP (28.33 ± 1.3), and SN (29.0 ± 2.9) in between 12.5 and 19.5 t/ha biochar dosages followed by the *Kekeba* (KEK) wheat variety (Figure 3). However, the *Kingbird* (KIN) variety had comparatively lower growth performance due to parthenium biochar additions in two growing years (Figure 3).

**Figure 4:**
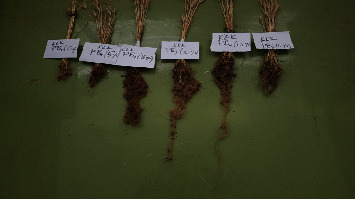
The root length of *Kekeba* wheat variety with increasing the biochar.

Root length, root dry biomass, and shoot dry biomass of wheat varieties also showed significant changes (*p* ≤ 0.001) between biochar dosages and wheat growing periods. Correspondingly, there was a considerable interaction effect between the concentration of biochar and wheat varieties in root length and root dry biomass) and shoot dry biomass (Table 8). However, the combined factors (biochar rates, wheat varieties, and cropping years) have no significant interaction effect on the agronomic parameters (Table 8). Wheat varieties KEK and AGO were comparatively higher overall average values in terms of SDB (12.0; 11.47), RDB (5.03; 5.0), and RL (35.5; 43.75), respectively, compared to the KIN wheat variety across the two cropping years (Figure 4).

## 4. Discussion

### 4.1. Effect of Biochar Addition on the Soil Physical Properties

The results of the study showed that an increase in the applied biochar dosage increased the clay content and decreased the sand fraction. Van-Zwieten et al. [22] demonstrated that biochar applied in the soil mixture could significantly influence soil texture and increase the clay fractions. However, there was no considerable difference across years and with the combined effect of the variables, which could be associated with the nature of parent material that cannot modify in two years. The notable difference in SMC of acidic soils was the result of biochar application that enhances the formation of large surface area and increases soil porosity, which increases the water holding capacity. This finding agreed with the work of Uzoma et al. [34], who stated that the amendment of soil by cow manure biochar lifts up SMC by 15%. Similarly, Asai et al. [35] and Jones et al. [36] reported the significant effect of biochar to increase the water holding capacity of degraded soils.

### 4.2. Effect of *P. hysterophorus* Biochar on the Chemical Characteristics of Acidic Soils

Biochar is known to decrease soil acidity and, in turn, enhance plant growth by increasing soil fertility [37]. Thus, in all treatments, application of *P. hysterophorus* biochar increased the soil pH by shifting from acidic (5.4) to neutral (6.7) and slightly alkaline (7.8) conditions (Table 5), which is an ideal soil pH environment for crop growth. The effect was due to the biochar having higher pH content (Table 1) and, in turn, a liming function capable of neutralizing acid-forming cations in the soil exchange sites [38] and the dissolution of alkaline carbonates and hydroxides minerals present mainly in the ash fraction of the biochar [12]. Previous studies on the rice husk biochar increased the acidic tea garden from soil pH 3.33 to 3.63 [39]; the chicken manure biochar indicated a considerable change in the soil pH from 3.9 to 5.1 [40], and when 39 t·ha^−1^, herbaceous feedstock applied in the soil alter the pH from 7.1 to 8.1 [17]. Due to the chemical composition of the char (Table 1), the soil EC has augmented within the biochar rates. However, irrespective of the EC values, the effect of salinity was found negligible in all soil types (Table 5, [41]). Mensah and Frimpong [42] indicated that the soil EC was increased by 10.8% in the 2.5 t/ha of tannery wastes biochar treatment. Despite these enhancing results, there is a study that reports the opposite for long-term field trials. Jones et al. [36] showed that three years of biochar addition to the soil system significantly (*p* < 0.05) reduced the soil EC from 46 to 43 *μ*s·cm^−1^ in a UK field trial. *P. hysterophorus* biochar-treated soils found in the ranges of between medium to a very high content of the exchangeable base and CEC [41]. These higher contents are ascribed to the potential effect of the pyrolysis carbon to release base-forming cations, which offset the incidence of soil acidity. Similar findings were reported by Agegnehu et al. [17], Agegnehu et al. [27], and Mensah and Frimpong [42] who found a highly variable charge in organic material that increases the exchangeable bases after the addition of biochar. Similar findings of an increase in soil CEC (from 7.41 to 10.8 cmol^+^/kg) and PBS (6.4 to 26%) in the biochar-treated soils were also reported by Bhattarai et al. [43]. Also, an increasing trend in Mg content compared to the control treatment, but with no considerable difference was observed in this study. And this might be related to the lower production temperature and the feedstock nature. In the same ways, no significant difference in the concentration of Mg content was due to the application of red oak biochar (500°C; 2%) reported by Mensah and Frimpong [42]. On the other hand, the addition of *P*. *hysterophorus* biochar (12.5–26.5 t/ha) to acidic soil improved Av. P and SOC contents by 5.5 and 2.9%, respectively, compared to the control (Table 5). This trend of an increase in Av. P and SOC contents are associated with the effects of biochar application. The result agreed with the findings of Jones et al. [36], who reported the effect of biochar in the improvement of total organic carbon from 2.27 to 2.78% and Av. P from 15.7 to 15.8 mg·kg^−1^. This was due to the biochar effect that increased the soil pH (Table 1) and surface area of soil colloids, which in turn increased the nutrient holding capacity of the soil. In similar studies, Agegnehu et al. [17] and Mensah and Frimpong [42] also found higher soil Av. P in biochar-amended soil due to an increase in soil pH.

### 4.3. Potential of Parthenium Biochar to Immobilize Soil Micronutrients

The availability of Fe, Mn, Zn, Cu, and Zn is generally higher at lower pH or acidic soil [44]. However, in this study, the addition of parthenium biochar in acidic soils reduced the bioavailability of micronutrients (Fe, Mn, Cu, and Zn) because the application of biochar in acidic soil can adsorb and immobilize heavy metals [11, 15]. Also, as *P. hysterophorus* biochar dosages increase, rates of micronutrients significantly decrease in the soil system. As a result, the proportion of heavy metals in biochar-treated soils was found in the range of low to medium compared with control as per the ratings of FAO [41]. In a pot trial study, the application of hardwood-derived biochar on contaminated soils also reduced the availability of zinc (Zn) and cadmium (Cd) metal types [13]. Similarly, Qiao et al. [16] described that the application of biochar in acidic soils significantly decreased acid-soluble heavy metal concentrations (Fe, Mn, Pb, and Cd) and their bioavailability for plants. Moreover, eigenvector coefficients and the PCA biplot (Table 7; Figure 2) showed a strong and positive association between the biochar dosages and years in improving essential soil nutrients (pH, basic cations, Av. P, and SOC), but micronutrients were negative loading with increasing biochar dosages and soil sampling years. Previous studies by Agegnehu et al. [27] indicated that most of the parameters (K, SOC, and CEC) equally contributed to the total variation and thus positive loading.

### 4.4. Performance of Wheat Varieties Influenced by *P. hysterophorus* Biochar

The application of biochar in the acidic soil exhibited a greater effect on plant height (PH) and head length (HL), especially in AGO and KEK wheat varieties as compared to the KIN variety (Figure 3). In contrast, the control treatment had to wane the agronomic performance of the studied wheat varieties, which may be due to the soil minerals are being chemically less available to the plants in acidic soil. But the higher growth performance in biochar-amended soils is the capacity of the char to lower soil acidity and improves the potential availability of essential nutrients for wheat growth. And this was in line with the findings of Mensah and Frimpong [42] who reported the increments of plant height, the number of leaves, and stems girth of two maize varieties after the application of corncob biochar alone or in combined with compost in the acidic rainforest and coastal savannah soils. However, the performances of the varieties decreased as the parthenium biochar dosages increased above 23 t/ha, which could be due to an increase in soil pH (>7.0) above the optimum level where wheat growth requires a pH of 6 to 7. A previous study by Asai et al. [35] revealed that the addition of higher than 16 t/ha of teak and rosewood biochar in the field condition of acidic soil reduces rice growth. The *P*. *hysterophorus* biochar significantly increases the radicle length, root dry biomass, shoot dry biomass, seed number per plant (SNPP), and spikelet number (SN) of the wheat varieties, which might be due to liming effect of basic cations available in the char (Table 1). An increasing trend in biomass agrees with the findings of Agegnehu et al. [17] and Bhattarai et al. [42]. However, Van-Zwieten et al. [22] observed that the wheat and soybean biomass production was not affected solely by the slow pyrolysis of paper mill biochar addition. Moreover, in this study, the AGO wheat variety observed the higher entire biomass in acidic soil amended by parthenium biochar, followed by the KEK variety, whereas the lower entire growth performance was recorded in KIN wheat variety with a similar amendment in acidic soils.

## 5. Conclusions and Future Perspectives

Soil acidity and invasive weeds significantly reduced crop productivity and sustainability. Thus, ameliorating soil acidity through averting noxious weed into a usable form of biochar is indispensable to boost fertility and crop productivity. The present study revealed that *P. hysterophorus* biochar considerably increased the SMC, pH, Av. P, exchangeable bases, and SOC compared to the control treatments (with no biochar). However, soil particle size fractions did not show significant variation across the years. *P. hysterophorus* biochar significantly decreased the bioavailability of metal contaminants that attributes to the occurrence of soil acidity. Furthermore, the higher agronomic performance of the wheat varieties found in the biochar-amended acidic soils. Also, the higher crop assay parameters were recorded in *Ogolcha* (AGO) and *Kekeba* (KEK) wheat varieties compared to the lower performance of the *Kingbird* (KIN) variety. However, the performances of the entire wheat varieties gradually declined as the concentration of chars exceeded over 23 t/ha. To this end, integrated agricultural inputs with the *P. hysterophorus* biochar are very crucial to amend soil acidity. More comprehensive studies will be needed to evaluate the effectiveness of *P. hysterophorus* biochar in the amelioration of acidic soil, soil microorganisms, and yields on field conditions of different soil types.

## Figures and Tables

**Table 1:** The chemical composition of the acidic soil sample and the parthenium biochar.

Chemical Properties	pH (H_2_O)	EC ms/cm	OC (%)	CEC (Meq/100 kg)	Ex. Ca (cmol^+^/kg)	Ex. K (cmol^+^/kg)	Av. P (ppm)
Acidic soil sample	5.4	0.12	0.8	20.2	11.5	0.6	13.8
Parthenium biochar	12.23	10.7	26.8	42.64	25.6	15.4	176.7

*Note.* pH, power of hydrogen; EC, electrical conductivity; OC, organic carbon; CEC, cation exchange capacity; Ex.Ca, exchangeable calcium; Ex. K, exchangeable potassium; and Av. P, available phosphorous.

**Table 2:** Results of the general linear model procedure, analyzing the effect of parthenium biochar dosages and soil sample years on the soil physical properties.

Sources of variations	DF	Sand (%)		Clay (%)		Silt (%)		SMC (%)	
		MS	P	MS	P	MS	P	MS	P
SSY	1	2.8	*p* > 0.05	2.2	*p* > 0.05	0.2	*p* > 0.05	0.035	*p* ≤ 0.05
PBD	5	15.2	*p* ≤ 0.01	7.1	*p* ≤ 0.01	4.2	*p* ≤ 0.05	30.06	*p* ≤ 0.01
SSY^*∗*^PBD	5	1.6	*p* > 0.05	1.2	*p* > 0.05	2.98	*p* > 0.05	0.40	*p* > 0.05
Error	24	0.69	—	0.69	—	1.42	—	0.3	—
Mean		58.4	—	22.02	—	19.7	—	19.13	—
*R*-square (*R*^2^)		0.84	—	0.72	—	0.52	—	0.95	—
Total	36	3412.4	—	486.6	—	390.0	—	370.32	—

*Note.* SSY, soil sample years; PBD, parthenium biochar dosages; MS, mean square; P, probability level; DF, degree of freedom, and SMC, soil moisture content.

**Table 3:** Effect of *P. hysterophorus* biochar amounts (*n* = 6) and years (*n* = 2) on the soil texture and SMC of acidic soils (mean ± SD).

Soil	Soil sample	Parthenium biochar rates						LSD
Parameters	Years	PB0% 0 t/ha	PB5.33% 12.5 t/ha	PB8.0% 16 t/ha	PB10.67% 19.5 t/ha	PB13.3% 23 t/ha	PB 16% 26.5 t/ha	
Sand	First year	60.61 ± 0.6^a^^*∗*^	57.6 ± 0.6^c^	58.9 ± 0.6^bc^	57.6 ± 1.1^c^	57.6 ± 0.5^c^	59.6 ± 0.6^a^	3.0
	Second year	61.9 ± 1.1^a^^*∗*^	57.3 ± 1.0^bc^	57.3 ± 1.0^bc^	56.9 ± 0.6^bc^	56.6 ± 0.6^c^	58.6 ± 0.6^b^	0.77
	Overall	61.3 ± 1.0^a^^*∗*^	57.5 ± 0.7^c^	58.1 ± 1.1^dc^	57.3 ± 0.8^c^	57.1 ± 0.7^c^	58.1 ± 0.9^b^	0.42

Clay	First year	20.2 ± 0.00^c^	23.5 ± 0.6^a^^*∗*^	22.8 ± 1.1^ab^	21.5 ± 1.1^bc^	22.2 ± 0.00^ab^	20.5 ± 0.6^c^	3.0
	Second year	21.2 ± 1.0^c^	22.5 ± 0.6^bc^	23.2 ± 1.0^ab^^*∗∗*^	23.2 ± 1.0^ab^^*∗∗*^	22.8 ± 0.6^ab^	20.8 ± 1.1^c^	2.3
	Overall	20.6 ± 0.8^b^	23.0 ± 0.7^a^^*∗*^	23.0 ± 0.9^a^^*∗*^	22.3 ± 1.3^a^	22.5 ± 0.5^a^	20.67 ± 0.8^b^	2.3

Silt	First year	19.2 ± 0.6	18.9 ± 0.6	18.5 ± 2.0	20.9 ± 2.3	20.2 ± 0.6	19.9 ± 1.1	NS
	Second year	17.2 ± 1.1^b^	20.2 ± 0.6^a^	20.2 ± 1.5^a^	19.9 ± 1.1^a^	20.5 ± 0.00^a^^*∗∗*^	20.5 ± 0.0^a^^*∗∗*^	0.77
	Overall	18.2 ± 1.3^b^	19.5 ± 0.9^ab^	19.4 ± 1.8^ab^	20.4 ± 1.7^a^	20.4 ± 0.4^a^	20.5 ± 0.0^a^^*∗∗*^	2.2

% SMC	First year	15.8 ± 0.3^e^	17.5 ± 0.4^cd^	18.99 ± 0.2^bc^	19.4 ± 0.8^b^	20.8 ± 0.8^a^	21.02 ± 0.4^a^^*∗*^	1.98
	Second year	15.1 ± 0.2^d^	17.99 ± 0.3^c^	18.8 ± 0.9^c^	20.1 ± 0.6^b^	20.99 ± 0.4^ab^	21.9 ± 0.1^a^^*∗*^	2.61
	Overall	15.5 ± 0.5^e^	17.7 ± 0.4^d^	19.4 ± 0.8^c^	19.8 ± 0.8^b^	20.9 ± 0.6^a^	21.6 ± 0.5^a^^*∗*^	1.82

Within rows, means followed by the same letter are not significantly different (*p* > 0.05); ^*∗*^significant at *p* ≤ 0.01; ^*∗∗*^significant at *p* ≤ 0.05; NS, not significant; LSD, least significant difference; % SMC, percent soil moisture content; PB; parthenium biochar; and SD, standard deviation.

**Table 4:** Summary of two-way ANOVA results for the soil chemical properties in terms of soil sample years and parthenium biochar dosages.

Source of variations	DF	pH (H_2_O)		pH (KCl)		EC (ms/cm)		Ca (cmol^+^/kg)		K (cmol^+^/kg)		Na (cmol^+^/kg)		Mg (cmol^+^/kg)		CEC(cmol^+^/kg)		AvP (ppm)		OC (%)	
		MS	P	MS	P	MS	P	MS	P	MS	P	MS	P	MS	P	MS	P	MS	P	MS	P
SSY	1	3.17	*p* ≤ 0.01	4.02	*p* ≤ 0.01	2.14	*p* ≤ 0.001	85.5	*p* ≤ 0.001	34.0	*p* ≤ 0.001	0.3	*p* ≤ 0.001	41.1	*p* ≤ 0.01	268.5	*p* ≤ 0.001	4285.7	*p* ≤ 0.001	5.2	*p* ≤ 0.001
PBD	5	4.9	*p* ≤ 0.001	4.04	*p* ≤ 0.001	1.6	*p* ≤ 0.001	25.9	*p* ≤ 0.001	35.7	*p* ≤ 0.001	0.2	*p* ≤ 0.001	8.9	p > 0.05	73.6	*p* ≤ 0.001	2561.7	*p* ≤ 0.001	1.84	*p* ≤ 0.001
SSY*∗*PBD	5	0.14	*p* > 0.05	0.05	*p* > 0.05	0.15	*p* ≤ 0.001	6.5	*p* ≤ 0.005	2.7	*p* > 0.05	0.009	*p* ≤ 0.005	2.5	*p* > 0.05	11.9	*p* ≤ 0.005	227.8	*p* ≤ 0.001	0.34	*p* ≤ 0.001
Error	24	0.26	—	0.28	—	1.015	—	1.11	—	11.16	—	0.002	—	2.6	—	2.3	—	19.8	—	0.0016	—
Mean		7.1	—	6.44	—	0.01	—	15.4	—	4.5	—	0.59	—	6.2	—	26.24	—	48.3	—	1.5	—
*R*-square (*R*^2^)		0.82	—	0.78	—	0.97	—	0.90	—	0.89	—	0.81	—	0.60	—	0.92	—	0.97	—	0.97	—

SSY, soil sample years; PBD, parthenium biochar dosages; MS, mean square; P, probability level; and DF, degree of freedom.

**Table 5:** The status of chemical properties in acidic soil into two soil sample years and parthenium biochar dosages (mean ± SD).

Soil parameters	Soil sample	Parthenium biochar rates						LSD
	Years	PB0% 0 t/ha	PB5.33% 12.5 t/ha	PB8.0% 16 t/ha	PB10.67% 19.5 t/ha	PB13.3% 23 t/ha	PB16% 26.5 t/ha	
pH (H_2_O)	First year	5.44 ± 0.12^b^	6.3 ± 0.5^ab^	6.7 ± 0.5^a^	7.1 ± 1.1^a^	7.5 ± 0.6^a^^*∗∗*^	7.3 ± 0.6^a^	2.06
	Second year	5.45 ± 0.14^d^	7.0 ± 0.2^c^	7.6 ± 0.5^b^	7.96 ± 0.2^ab^	8.1 ± 0.2^a^^*∗*^	8.1 ± 0.15^a^	1.58
	Combined	5.44 ± 0.12^c^	6.7 ± 0.^5b^	7.2 ± 0.7^ba^	7.55 ± 0.8^a^	7.8 ± 0.5^a^^*∗∗*^	7.8 ± 0.5^a^	1.74

pH (KCl)	First year	4.8 ± 0.05^b^	5.64 ± 0.5^a^	6.1 ± 0.6^a^	6.4 ± 1.0^a^	6.8 ± 0.6^a^^*∗∗*^	6.8 ± 0.6^a^	2.01
	Second year	5.2 ± 0.7^c^	6.3 ± 0.1^b^	6.9 ± 0.5^a^	7.3 ± 0.13^a^	7.4 ± 0.1^a^^*∗∗*^	7.4 ± 0.1^a^	1.78
	Combined	5.0 ± 0.5^c^	5.99 ± 0.5^b^	6.5 ± 0.7^ab^	6.9 ± 0.8^a^	7.1 ± 0.5^a^	7.1 ± 0.5^a^^*∗∗*^	1.55

EC (ms/cm)	First year	0.09 ± 0.05^d^	0.63 ± 0.04^c^	0.76 ± 0.07^c^	0.8 ± 0.2^bc^	0.97 ± 0.16^b^	1.34 ± 0.2^a^^*∗*^	0.58
	Second year	0.15 ± 0.05^d^	0.8 ± 0.15^c^	1.37 ± 0.07^b^	1.62 ± 0.1^a^	1.75 ± 0.11^a^	1.85 ± 0.1	0.57
	Combined	0.12 ± 0.04^d^	0.71 ± 0.14^c^	1.1 ± 0.34^b^	1.2 ± 0.5^b^	1.36 ± 0.4^a^	1.6 ± 0.3^a^^*∗*^	0.35

Ca (cmol^+^/kg)	First year	11.95 ± 0.9^c^	12.9 ± 0.3^bc^	14.15 ± 0.3^ab^	15.12 ± 1.8^a^	13.9 ± 0.7^ab^	14.9 ± 1.0^a^^*∗∗*^	3.17
	Second year	11.14 ± 0.3^c^	15.9 ± 0.6^b^	17.9 ± 1.4^ab^	18.45 ± 0.9^a^	19.32 ± 1.5^a^^*∗*^	18.7 ± 1.2^a^	4.8
	Combined	11.54 ± 0.7^c^	14.4 ± 1.7^b^	16.01 ± 2.2^ab^	16.8 ± 2.2^a^	16.6 ± 3.1^a^	16.8 ± 2.2^a^^*∗∗*^	4.4

K (cmol^+^/kg)	First year	0.6 ± 0.05^b^	1.75 ± 0.7^b^	3.9 ± 1.5^a^	5.3 ± 2.0^a^^*∗∗*^	5.2 ± 1.5^a^	4.2 ± 0.6^a^	4.63
	Second year	0.72 ± 0.08^d^	3.1 ± 0.5^c^	5.3 ± 0.6^b^	7.4 ± 1.0^a^	7.8 ± 1.0^a^	8.3 ± 1.1^a^^*∗*^	4.3
	Combined	0.64 ± 0.1^d^	2.4 ± 0.9^c^	4.6 ± 1.3^b^	6.4 ± 1.8^a^	6.5 ± 1.8^a^^*∗*^	6.2 ± 2.3^a^	3.8

Na (cmol^+^/kg)	First year	0.21 ± 0.04^c^	0.81 ± 0.2^b^	0.5 ± 0.09^a^	0.6 ± 0.04^ab^	0.45 ± 0.1^ac^^*∗*^	0.43 ± 0.03^ac^	0.6
	Second year	0.24 ± 0.05^c^	0.6 ± 0.08^b^	0.74 ± 0.12^ab^	0.8 ± 0.06^ab^	0.92 ± 0.2^a^^*∗*^	0.83 ± 0.2^ab^	0.53
	Combined	0.22 ± 0.04^b^	0.7 ± 0.2^a^^*∗*^	0.62 ± 0.1^a^	0.7 ± 0.1^a^^*∗*^	0.7 ± 0.3^a^^*∗*^	0.6 ± 0.2^a^	0.39

Mg (cmol^+^/kg)	First year	3.99 ± 0.9	5.4 ± 1.5	5.8 ± 2.0	5.0 ± 2.8	5.99 ± 1.3	4.8 ± 1.1	NS
	Second year	3.8 ± 0.5^b^	7.4 ± 1.5^a^	8.7 ± 0.6^a^	8.5 ± 1.2^a^	7.9 ± 2.2^a^^*∗∗*^	7.6 ± 1.9^a^	4.75
	Combined	3.9 ± 0.7	6.4 ± 1.8	7.3 ± 2.0	6.8 ± 2.7	6.9 ± 1.9	6.2 ± 2.0	NS

OC (%)	First year	0.8 ± 0.09^d^	0.8 ± 0.1^d^	1.1 ± 0.08^c^	1.22 ± 0.04^c^	1.47 ± 0.1^b^	1.71 ± 0.1^a^^*∗*^	0.42
	Second year	0.77 ± 0.1^d^	1.4 ± 0.15^c^	1.9 ± 0.07^b^	2.2 ± 0.12^b^	2.8 ± 0.2^a^^*∗*^	2.6 ± 0.1^a^	0.52
	Combined	0.8 ± 0.1^d^	1.1 ± 0.3^c^	1.5 ± 0.4^b^	1.7 ± 0.5^b^	2.1 ± 0.7^a^	2.2 ± 0.5^a^^*∗*^	0.63

Av. P (ppm)	First year	13.6 ± 1.9^e^	24.2 ± 1.5^dc^	32.6 ± 3.4^c^	38.8 ± 9.4^bc^	49.2 ± 3.2^b^	65.8 ± 7.5^a^^*∗*^	24.9
	Second year	14.06 ± 1.5^c^	58.1 ± 2.5^b^	59.8 ± 2.9^b^	62.7 ± 5.0^b^	79.7 ± 2.8^a^	80.7 ± 3.9^a^^*∗*^	16.9
	Combined	13.8 ± 1.5^e^	41.1 ± 18.6^d^	46.1 ± 15.1^cd^	50.78 ± 14.7^c^	64.4 ± 16.9^b^	73.2 ± 9.7^a^^*∗*^	13.6

CEC (cmol^+^/kg)	First year	19.7 ± 1.3^b^	21.1 ± 0.9^b^	24.9 ± 2.1^a^	24.8 ± 1.4^a^	25.6 ± 0.9^a^^*∗∗*^	24.6 ± 2.5^a^	5.9
	Second year	20.6 ± 0.9^d^	27.1 ± 1.5^c^	28.3 ± 1.0^b^	31.8 ± 1.0^a^	32.7 ± 1.8^a^	33.2 ± 1.7^a^^*∗∗*^	5.5
	Combined	20.2 ± 1.1^c^	24.1 ± 3.4^b^	26.6 ± 2.4^a^	28.4 ± 3.9^a^	29.2 ± 4.0^a^^*∗∗*^	28.9 ± 5.0^a^	4.8

Within rows, means followed by the same letter are not significantly different (*p* > 0.05); ^*∗*^significant at *p* ≤ 0.01; ^*∗∗*^ significant at *p* ≤ 0.05; NS, not significant; LSD, least significant difference; PB; parthenium biochar; SD, standard deviation.

**Table 6:** Multivariate analysis for the soil micronutrients contents related with the soil sample years and parthenium biochar dosages.

Sources of variations	DF	Cu (mg/L)		Zn (mg/L)		Mn (mg/L)		Fe (mg/L)	
		MS	P	MS	P	MS	P	MS	P
SSY	1	0.94	*p* < 0.05	0.02	*p* > 0.05	5.4	*p* ≤ 0.001	14.7	*p* < 0.05
PBD	5	1.67	*p* ≤ 0.001	0.69	*p* ≤ 0.001	3.5	*p* ≤ 0.001	45.6	*p* ≤ 0.001
SSY*∗*PBD	5	0.19	*p* > 0.05	0.007	*p* > 0.05	0.33	*p* < 0.05	0.75	*p* > 0.05
Error	24	0.12	—	0.018	—	0.1	—	2.9	—
Mean	0.42	—	0.74	—	2.4	—	8.1	—	—
*R*-square (*R*^2^)	0.78	—	0.89	—	0.90	—	0.77	—	—

SSY, soil sample years; PBD, parthenium biochar dosages; MS, mean square; P, probability level; DF, degree of freedom.

**Table 7:** Eigenvector coefficients for the soil chemical properties contained in the first sixth principal components (PC1–6) derived from the principal component analysis of the indicated parameters in six treatments over two years.

Soil parameters	PC1	PC2	PC3	PC4	PC5	PC6
pH (H_2_O)	**0.285**	0.0163	−**0.251**	0.215	0.018	0.313
pH (KCl)	0.282	−0.0028	−**0.252**	0.238	0.039	0.329
EC	**0.294**	0.012	0.217	0.013	0.024	0.108
Av. P	**0.289**	−**0.0176**	0.222	0.131	−0.027	−0.114
OC	0.282	0.159	0.332	0.062	−0.135	0.050
CEC	**0.291**	0.252	0.109	0.081	0.043	−0.126
Ca^++^	0.283	0.328	0.037	0.046	0.126	−0.0014
K^+^	0.272	−**0.005**	**0.428**	0.039	0.054	0.154
Na^+^	0.224	0.109	0.0024	−**0.880**	0.266	−0.005
Mg^++^	0.224	**0.449**	−**0.631**	−0.0271	−0.090	−0.133
Cu	−00.236	0.443	0.171	−0.172	−**0.545**	**0.512**
Zn	−0**0.251**	**0.463**	0.133	0.101	−0.165	−**0.398**
Mn	−**0.271**	0.059	−0.0471	−0.022	0.331	**0.537**
Fe	−0.239	0.418	0.151	0.223	**0.667**	0.007
Eigen value	10.407	0.811	0.677	0.537	0.417	0.314
% variance	0.743	0.058	0.048	0.038	0.03	0.022

Bold values indicate the most and the least explained variables.

**Table 8:** Results of three-way ANOVA showing the effect of *P. hysterophorus* biochar on the agronomic performance of wheat varieties across cropping years.

Sources	DF	PH		SNPP		HL		SN		RL		RDB		SDB	
Factors		MS	P	MS	P	MS	P	MS	P	MS	P	MS	P	MS	P
Years (*Y*)	1 108.00	*p* ≤ 0.05	205.56	*p* ≤ 0.001	0.083	*p* > 0.05	10.7	*p* > 0.05	3616.9	*p* ≤ 0.001	51.4	*p* ≤ 0.001	75.0	*p* ≤ 0.001	
Varieties (*V*)	2	189.7	*p* ≤ 0.001	153.0	*p* ≤ 0.001	4.37	*p* ≤ 0.001	131.4	*p* ≤ 0.001	616.4	*p* > 0.05	38.1	*p* ≤ 0.001	2.4	*p* > 0.05
Biochar dose (BD)	5	102.03	*p* ≤ 0.05	144.12	*p* ≤ 0.001	2.75	*p* ≤ 0.001	103.64	*p* ≤ 0.001	3155.8	*p* ≤ 0.001	35.0	*p* ≤ 0.001	51.9	*p* ≤ 0.001
Y X BD	5	18.8	*p* > 0.05	16.0	*p* ≤ 0.05	0.44	*p* > 0.05	26.73	*p* ≤ 0.001	516.3	*p* > 0.05	0.6	*p* > 0.05	8.1	*p* > 0.05
Y X V	2	41.2	*p* > 0.05	2.06	*p* > 0.05	0.13	*p* > 0.05	1.59	*p* > 0.05	0.44	*p* > 0.05	0.15	*p* > 0.05	40.5	*p* ≤ 0.005
BDXV	10	75.26	*p* ≤ 0.05	32.5	*p* ≤ 0.001	1.62	*p* ≤ 0.001	58.69	*p* ≤ 0.001	687.6	*p* ≤ 0.05	9.1	*p* ≤ 0.005	17.7	*p* ≤ 0.005
Y X V XBD	10	7.6	*p* > 0.05	3.7	*p* > 0.05	0.28	*p* > 0.05	4.81	*p* > 0.05	52.2	*p* > 0.05	0.23	p > 0.05	3.3	*p* > 0.05
Model	35	57.2	*p* ≤ 0.05	47.98	*p* ≤ 0.01	1.26	*p* ≤ 0.01	44.67	*p* ≤ 0.01	879.06	*p* ≤ 0.001	11.4	*p* ≤ 0.001	19.2	*p* ≤ 0.001
*R* ^2^ (*R*-square)		0.52	—	0.81	—	0.72	—	0.86	—	0.61	—	0.64	—	0.60	—
Error	72	25.8	—	5.44	—	0.23	—	3.52	—	283.6	—	3.1	—	6.1	—
Total	107	36.07	—	19.4	—	0.57	—	16.98	—	478.4	—	5.833	—	10.41	—

*Note.* DF, degree of freedom; PH, plant height; HL, head length; SNPP, seed number per plant, SN, spikelet number; RL, root length; RDB, root dry biomass; SDB, shoot dry biomass.

## Data Availability

The raw data sets used and/analyzed during the current study are available from the corresponding author on reasonable request.

## Supplementary Materials

Supplementary Materials Supplementary 1: framework of the *P. hysterophorus* biochar preparation. Supplementary 2: conceptual framework of the soil sample preparation and layout of the research design for cropping.
