# Supplementary material for: Evaluating Slow Pyrolysis of Parthenium hysterophorus Biochar: Perspectives to Acidic Soil Amelioration and Growth of Selected Wheat (Triticum aestivum) Varieties
Source: ScientificWorldJournal. 2022 Jan 4;2022:8181742. doi: 10.1155/2022/8181742 (PMC8752242; doi:10.1155/2022/8181742)
Supplement: Supplementary Materials — Supplementary 1: framework of the P. hysterophorus biochar preparation. Supplementary 2: conceptual framework of the soil sample preparation and layout of the research design for cropping. [file 8181742.f1.docx]

**
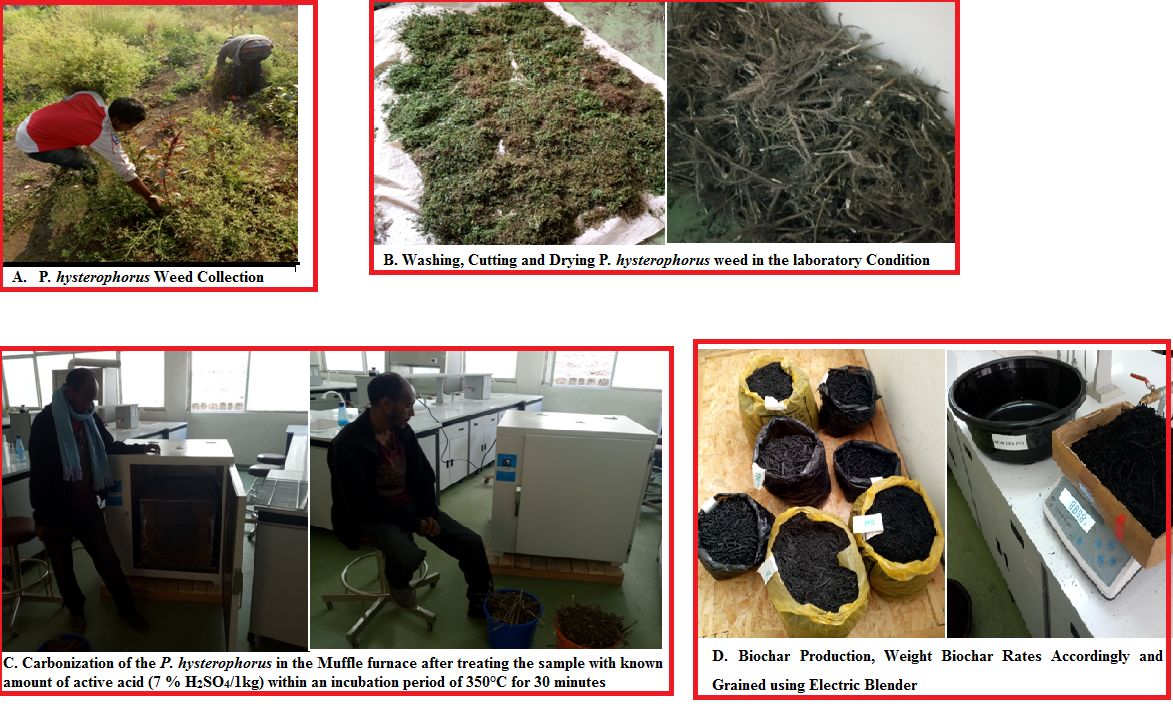
Supplementary 1 Frame work of the *P. hysterophorus* biochar preparation**

**
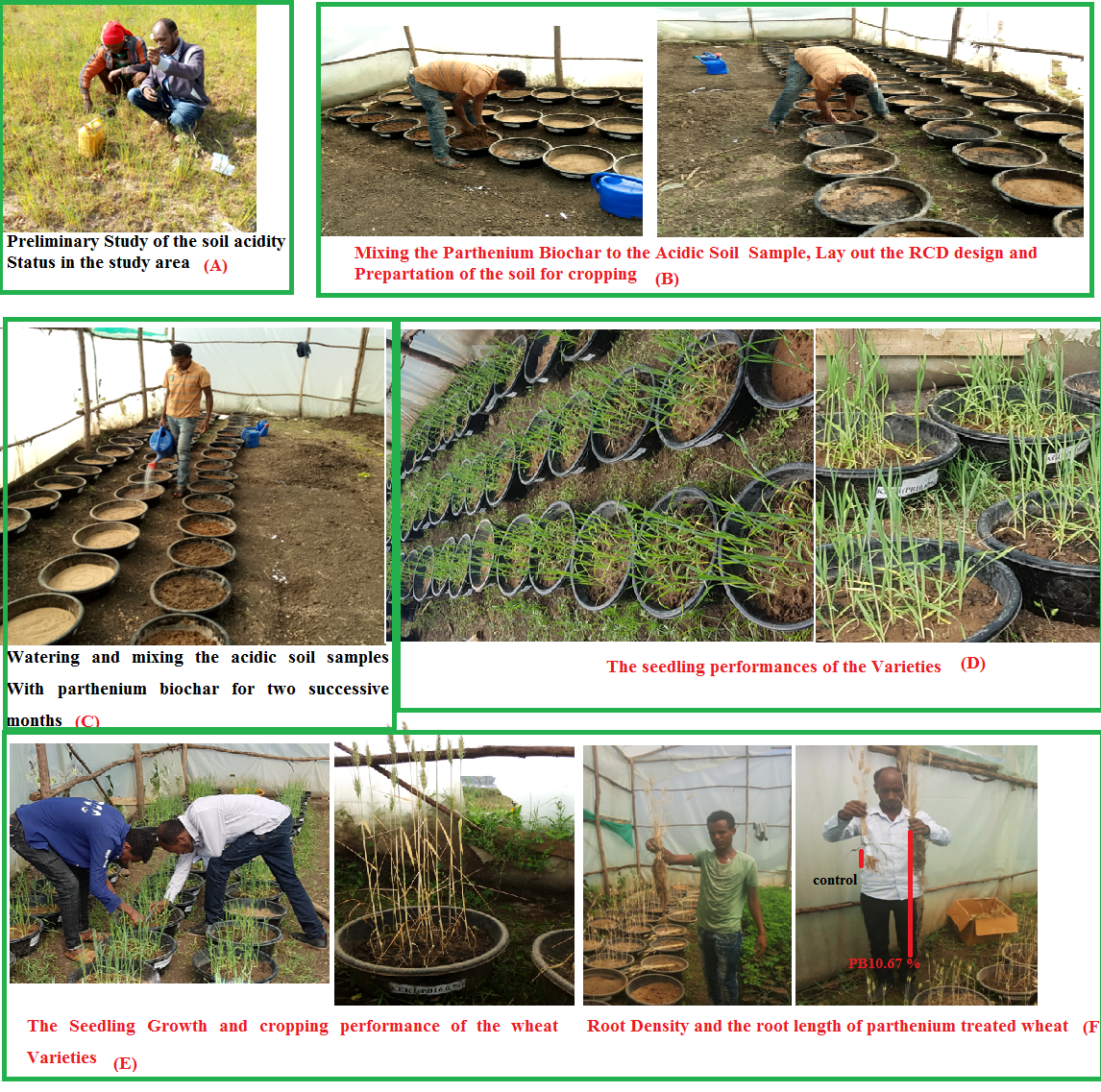
Supplementary 2 Conceptual framework of the soil sample preparation and lay-out the research design for cropping**
